# Study on the *In Vitro* Activity of Five Disinfectants against Nosocomial Bacteria

**DOI:** 10.3390/ijerph16111895

**Published:** 2019-05-29

**Authors:** Maria Teresa Montagna, Francesco Triggiano, Giovanna Barbuti, Nicola Bartolomeo, Osvalda De Giglio, Giusy Diella, Marco Lopuzzo, Serafina Rutigliano, Gabriella Serio, Giuseppina Caggiano

**Affiliations:** Department of Biomedical Science and Human Oncology, University of Bari “Aldo Moro”, Piazza G. Cesare 11, 70124 Bari, Italy; francescotrigg@hotmail.it (F.T.); giovanna.barbuti@uniba.it (G.B.); nicola.bartolomeo@uniba.it (N.B.); osvalda.degiglio@uniba.it (O.D.G.); giusy.diella@uniba.it (G.D.); marcolopuzzo@gmail.com (M.L.); serafina.rutigliano@uniba.it (S.R.); gabriella.serio@uniba.it (G.S.); giuseppina.caggiano@uniba.it (G.C.)

**Keywords:** disinfection, nosocomial bacteria, antimicrobial agent, *Klebsiella pneumoniae*, *Pseudomonas aeruginosa*, *Staphylococcus aureus*, *Enterococcus faecalis*

## Abstract

Nosocomial infections cause significant morbidity and mortality worldwide, and the pathogenic organisms responsible for such infections can develop resistance to antimicrobial agents. Understanding the activity of disinfectants against clinical and environmental bacterial isolates is therefore crucial. We analysed the in vitro activity of five antimicrobial products (phenolic compounds, didecyldimethylammonium chloride (DDAC), sodium hypochlorite, isopropanol + ammonium compounds (IACs), hydrogen peroxide) against 187 bacterial strains comprising clinical isolates, as well as 30 environmental isolates of *Pseudomonas aeruginosa* from hospital water samples. Disk diffusion assays were employed to assess antimicrobial activity. Hydrogen peroxide was significantly more active (*p* < 0.0001) than the other disinfectants against all *P. aeruginosa*, *Klebsiella pneumoniae*, *Enterococcus faecalis* and *Staphylococcus aureus* strains. It was also the only disinfectant with activity against both clinical and environmental strains of *P. aeruginosa*. DDAC and IAC-based disinfectants were ineffective against Gram-negative strains, but showed significant activity (particularly IACs, *p* < 0.0001) against the Gram-positive strains. Compared with IACs, DDAC was significantly more active on *E. faecalis* and less active on *S. aureus* (*p* < 0.0001). Sodium hypochlorite and phenol compounds, by contrast, were inactive against all bacterial strains. The development of disinfection procedures that are effective against all microorganisms is essential for limiting the spread of nosocomial infections.

## 1. Introduction

Disinfection procedures represent the main type of intervention in hospital settings against pathogenic or potentially pathogenic microorganisms [1,2] and aim to prevent or reduce complications due to infectious organisms. Surface contamination, in particular, is a public health problem, as healthcare-associated infections (HAIs) represent a significant cause of morbidity and mortality worldwide [3]. HAIs develop during hospitalization and a range of different microorganisms are frequently isolated [4] including multidrug-resistant bacteria, such as vancomycin-resistant enterococci (VRE), *Clostridium difficile, Pseudomonas aeruginosa, Acinetobacter* spp. and methicillin-resistant *Staphylococcus aureus* (MRSA) [5,6,7]. In particular, *S. aureus* and *P. aeruginosa* are hazardous microorganisms, able to grow on hard non-porous surfaces (e.g., metal pipes and floor drains) and develop biofilms that protect them from adverse conditions [8,9]. Their resistance to various antimicrobial substances can compromise patients’ therapeutic protocol [10], increasing the need to improve disinfection procedures in healthcare settings [11,12].

There are multiple factors contributing to the spread of HAIs, which include healthcare-associated factors (invasive device use, surgical procedures, inappropriate use of antimicrobial therapy), environmental factors (contaminated air-conditioning systems) and patient-related factors (severity of underlying illnesses, use of immunosuppressive agents, prolonged hospital stays) [13].

According to data reported by the European Centre for Disease Prevention and Control (ECDC), every year about 33,000 people in the European Union die from infections due to multidrug-resistant bacteria, most of them contracted in hospitals [14]. In Italy, the probability of contracting an infection during hospitalization is 6%, with 530,000 cases every year [15].

During the years of 2015–2017, the government of the Apulia region (in southern Italy) promoted the IM.P.A.C.T. Project (IMproving the health of PAtients supporting dynamiC healTh system and new technologies), a regional study focused on evaluating environmental microbial contamination in operating theatres to identify the most important contributory factors [16]. Among the various topics addressed, a cognitive survey on the type of disinfectant most often employed to sanitize surfaces highlighted the use of chlorine compounds (39.7%), quaternary ammonium compounds (24.4%), phenol compounds (16.7%), alcohol compounds (14.7%) and hydrogen peroxide (4.5%).

The aim of this study was to investigate the in vitro antibacterial activity of five disinfectants most frequently used in Apulian hospital practice (phenolic compounds, quaternary ammonium compounds, sodium hypochlorite, alcohol compounds, hydrogen peroxide), to assess the importance of choosing disinfection methods to be applied in healthcare facilities. Hydrogen peroxide was the only disinfectant found to be active against all of the tested microorganisms.

## 2. Materials and Methods

Five disinfectants were subjected to in vitro sensitivity tests using the agar diffusion method. They were supplied by hospital pharmacies and contained the following active substances:

1) o-phenylphenol (11.4%), p-ter-amylphenol (2.3%), o-benzyl-p-chloro-phenol (+8.2%);
2) didecyldimethylammonium chloride (8.5%) (DDAC);
3) sodium hypochlorite (1.1% active chlorine);
4) isopropanol (17.2%) + ammonium compounds (0.28%) (IACs);
5) hydrogen peroxide (1.5%).

According to the manufacturer’s instructions, the disinfectant based on phenol compounds was used by diluting the stock solution with sterile distilled water to 0.4%, the disinfectant based on DDAC was diluted to 2% and sodium hypochlorite was diluted to 5%. The other disinfectants were used at ready-to-use concentrations.

A total of 187 bacteria, stored in glycerol at −80 °C, were selected from stock cultures at the Laboratory of Hygiene at the University of Bari Aldo Moro. All microorganisms were isolated from recovered hospitalized patients, with the exception of 30 *P. aeruginosa* strains isolated from the hospital water network. In particular, the following microorganisms were tested:

- *Klebsiella pneumoniae*: 58 strains, of which 30 were susceptible to carbapenems (KP) and 28 were resistant to carbapenems (KPC);
- *Pseudomonas aeruginosa*: 59 strains, of which 30 were isolated from hospital water samples;
- *Staphylococcus aureus*: 40 strains, of which 20 were susceptible to methicillin (MSSA) and 20 were resistant to methicillin (MRSA);
- *Enterococcus faecalis*: 30 strains.

All strains were seeded onto Petri plates containing brain–heart infusion agar (BHIA; Biokar Diagnostics, Beauvais, France) to ensure vitality and purity. After incubation at 36 °C ± 1 °C for 24 h, a single colony was subcultured on triple sugar iron agar (TSI, Biolife Srl, Milano, Italy) and incubated at 36 °C ± 1 °C for 24 h. Before proceeding, *P. aeruginosa* strains were serologically typed by the in vitro agglutination test, using 16 different monovalent antisera (BioRad, Marmes-la Coquette, France).

To set up the agar diffusion test, the microorganisms were suspended in sterile saline solution to a final concentration of 0.5 McFarland (1.5 × 10^8^ colony forming units/ml) and evenly spread onto tryptic soy agar (TSA, VWR International Srl, Milano, Italy) using the rolling technique and sterile swabs. Meanwhile, sterile paper disks (Ø = 6 mm) were soaked with 10 µl of each disinfectant, left to dry for 1 min, and were then placed onto the surface of each inoculated plate. The plates were maintained for 1 h at room temperature, and were then incubated at 36 °C ± 1 °C for 24 h.

The effectiveness of each disinfectant was evaluated by measuring the diameter of the microbial inhibition zone around the paper disks. The microorganisms were considered sensitive when the inhibition zone diameter was >8 mm overall. Paper disks were removed to assess the presence or absence of growth below. Each test was repeated three times, and the results evaluated. Control tests were included in each phase by including standard strains, i.e., *P. aeruginosa* (ATCC 27853), *K. pneumoniae* (ATCC 43816), *E. faecalis* (NCTC 775) and *S. aureus* (NCTC 6571). Positive control plates were set up under identical incubation conditions, while a paper disk soaked with 10 µL of sterile saline was used as a negative control. All assays were carried out under aseptic conditions.

### Statistical Analysis

The Shapiro–Wilk test was used to determine whether the inhibition zone showed a normal distribution and consequently it was expressed as the median and interquartile range (IQR). The non-parametric Friedman’s test and the signed rank Wilcoxon test for pairwise comparison were used to compare the effects of antimicrobial products on each bacterial strain. The non-parametric Kruskal–Wallis test and Mann–Whitney test for pairwise comparisons were employed to compare the efficacy of each product tested between different bacterial strains. All tests of statistical significance were two-tailed, and *p*-values of less than 0.05 were considered statistically significant. All *p*-values obtained by pairwise comparisons were adjusted for multiplicity using the linear step-up method of Benjamini and Hochberg. Statistical analysis was performed using the software SAS (version 9.4 for PC, SAS Institute Inc, Cary, NC, USA).

## 3. Results

A total of 187 Bacteria belonging to various species (*P. aeruginosa, K. pneumoniae, E. faecalis and S. aureus*) were studied to verify the sensibility to five disinfectants.

*P. aeruginosa* O6 (25.4%), P O11 (22%), P O3 (20.3%) and P O4 (11.9%) were the most frequent serotypes detected (Table 1), with no differences detected in the distribution between clinical and environmental strains.

Table 2 shows the sizes of the inhibition zones generated by the different disinfectants. The product based on hydrogen peroxide was significantly more active (*p* < 0.0001) on all *P. aeruginosa*, *K. pneumoniae*, *E. faecalis* and *S. aureus* strains. No differences were observed between the clinical and environmental strains of *P. aeruginosa*.

IACs were significantly active (*p* < 0.0001) on *S. aureus* (MSSA and MRSA) and *E. faecalis*, but not on *P. aeruginosa* and *K. pneumoniae* (KP and KPC).

DDAC was also active against *S. aureus* (MSSA and MRSA) and *E. faecalis*, but inactive against all Gram-negative bacteria tested (Table 2). When compared with IACs, DDAC was significantly more active on *E. faecalis* and less active on *S. aureus* (*p* < 0.0001).

**Figure 1:**
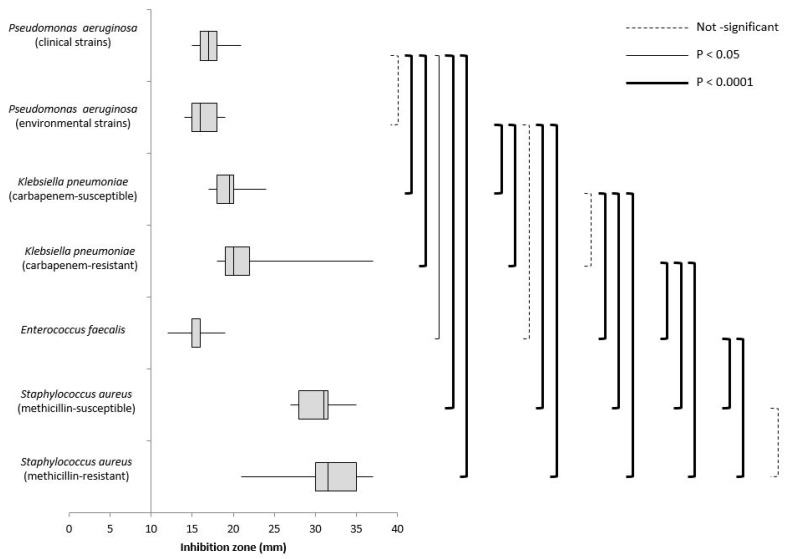
Box plot of the hydrogen peroxide effect. Pairwise multiple comparisons between different bacterial strains are shown.

Figure 1 shows the pairwise multiple comparisons of the hydrogen peroxide effect on different bacterial strains. No statistically significant differences were observed among clinical and environmental *P. aeruginosa* strains (*p* = 0.274) or among different serotypes, or between environmental *P. aeruginosa* and *E. faecalis* strains (*p* = 0.109). Furthermore, no differences were detected between carbapenem-susceptible and -resistant strains (KP vs. KPC, *p* = 0.075), or between methicillin-susceptible and -resistant strains (MSSA vs. MRSA, *p* = 0.069). All other pairwise multiple comparisons were statistically significant.

No statistically significant difference was observed between *E. faecalis* and *S. aureus* with DDAC (*p* = 1.000), or between MSSA and MRSA with IACs (*p* = 1.000) or DDAC (*p* = 0.106).

## 4. Discussion

In recent years, standardised protocols have been developed for cleaning and disinfection of the environment in healthcare settings [17,18,19,20,21,22,23]. Some researchers [24,25] have shown that a biocide can interact differently with microorganisms, either acting on the surface, the bacterial cell wall, the outer membrane or penetrating the cell, where it causes reversible or irreversible changes, acting on nucleic acids, inhibiting enzymes and cell growth. There is evidence in the literature that bacteria can develop resistance to disinfectants based on phenol, chlorine and alcohol compounds [26,27], whereas there is no clear evidence of resistance to quaternary ammonium compounds, including DDAC [26,28]. The use of appropriate antimicrobial agents is therefore a crucial step in good hospital practice because ineffective disinfection may increase the risk of transmission and horizontal spread of pathogens [29].

Our study analysed the in vitro activity of five antimicrobial products (phenolic compounds, DDAC, sodium hypochlorite, IACs, hydrogen peroxide), provided by hospital pharmacies, that are usually used in Apulian healthcare settings to target nosocomial strains. We also studied *P. aeruginosa* of environmental origin, in particular from hospital water samples, because it often colonizes the water network and is notoriously resistant to various treatments and therefore represents an important cause of infection for high-risk patients or those forced to stay for long periods in hospital. We also wanted to verify if the different *P. aeruginosa* serotypes influence the effectiveness of disinfectants. It has been reported that the mortality and predicted mortality rates are similar in patients infected by serotypes O1, O10 and O11, whereas the mortality rate is lower than the predicted mortality rate in patients infected by serotypes O6 and O2 [30]. Among the strains used in our study, *P. aeruginosa* serotypes O3, O6 and O11 were predominant. When evaluating the activity of hydrogen peroxide, no significant differences were observed between the different serotypes. The literature shows that the prevalence of *P. aeruginosa* serotypes varies from one hospital to another and from one country to another, with O6 and O11 being the most prevalent serotypes overall [31,32,33,34].

Hydrogen peroxide was the only disinfectant found to be active against all of the tested microorganisms. The biocidal effect of hydrogen peroxide is attributable to the -OH radical, formed by decomposition of the peroxide in the presence of catalysts, such as iron and copper ions, commonly found in microorganisms. The radical acts via an oxidative mechanism against the membrane, DNA and other cellular constituents of the microorganism [35]. Our results were in agreement with those of other authors [23,36,37,38] who reported the effectiveness of hydrogen peroxide against a wide range of microorganisms, including MRSA, VRE, *Mycobacterium tuberculosis*, spores, viruses and multidrug-resistant Gram-negative bacilli.

Regarding the other tested disinfectants, data in the literature show that chlorine compounds have a broad spectrum of antimicrobial activity, do not leave toxic residues, are unaffected by water hardness, remove dried or fixed organisms and biofilms from surfaces and are inexpensive and fast-acting [39,40]. Furthermore, phenol compounds are generally highly active, exerting bactericidal, fungicidal, virucidal and tuberculocidal activity [41]. In high concentrations, they act as gross protoplasmic poisons, penetrating and disrupting the cell wall and precipitating cellular proteins. Low concentrations of phenol and high molecular-weight phenol derivatives cause bacterial death by inactivation of essential enzyme systems and leakage of essential metabolites from the cell wall [41]. It is probable that using the products in accordance with the manufacturer’s guidelines means that the final concentration of the active components is too low to be effective against nosocomial strains. In fact, other studies [12,42] report higher concentrations of disinfectants based on sodium hypochlorite and phenol compounds compared with concentrations of the same products used in this study period.

DDAC is a mixture of alkyl-quaternary ammonium salts with typical alkyl chain lengths of C8, C10 and C12, commonly used in controlling contamination in healthcare facilities, veterinary practices and food manufacturing facilities, because of its high antimicrobial efficacy and its low irritation, corrosiveness and toxicity [43,44]. Gram-negative bacteria, in particular *Pseudomonas* spp. and *K. pneumoniae*, are known to be less susceptible to DDAC than Gram-positive bacteria, having generally high intrinsic resistance [45,46]. In accordance with the literature, our study showed that DDAC was inactive against all Gram-negative strains investigated but showed good activity against all Gram-positive strains (MRSA, MSSA and *E. faecalis*).

Alcohols, which included the two water-soluble chemical compounds, namely ethyl alcohol and isopropyl alcohol, are reported to be bactericidal against vegetative forms of bacteria, as well as being tuberculocidal, fungicidal and virucidal, but do not destroy bacterial spores [22,47]. Moreover, alcohols have excellent in vitro activity against resistant pathogens, such as MRSA and VRE strains [35], due to the denaturation of bacterial proteins. In accordance with the literature, our results showed that IAC-based disinfectants had good activity against Gram-positive bacteria (MRSA, MSSA, *E. faecalis*), although this activity was inferior to that of hydrogen peroxide, and had no activity against Gram-negative bacteria.

This study has some limitations, such as a limited number of tested microorganisms and the use of a single method, and thus future studies would ideally analyse an increased number of strains, evaluate different analytical methods and monitor the disinfectant activity over time.

## 5. Conclusions

Our findings showed that only hydrogen peroxide was active against both clinical and environmental bacterial strains. IACs and DDAC were active only against Gram-positive bacteria, whereas sodium hypochlorite and phenol compounds showed no activity. Since the effectiveness of a disinfection procedure depends on many factors (e.g., contact time, final concentration of the active ingredient, shelf-life of the product, temperature), accurate protocols in routine environmental reclamation practices are essential to improve the cleanliness of the hospital environment. The conscious use of a disinfectant in terms of choice and appropriate method of use represents a basic element in HAI prevention.

## Figures and Tables

**Table 1:** Serotypes of *Pseudomonas aeruginosa* among the clinical and environmental strains used in this study.

Serotype	*P. aeruginosa Strains*		
	Clinical	Environmental	Total (%)
O1	4	0	4 (6.8)
O3	6	6	12 (20.3)
O4	3	4	7 (11.9)
O6	6	9	15 (25.4)
O7	1	1	2 (3.4)
O9	2	2	4 (6.8)
O10	1	1	2 (3.4)
O11	7	6	13 (22.0)
Total	30 (50.8%)	29 (49.2%)	59 (100%)

**Table 2:** Median and interquartile range (IQR) of the inhibition zones (mm) generated by the products tested against *all tested bacteria* and standard microorganism strains.

*Microorganisms*		INHIBITION ZONE (mm)				
		Products Tested				
		Phenol Compounds	Sodium Hypochlorite	Hydrogen Peroxide	IACs	DDAC
***Pseudomonas aeruginosa***	Clinical (*n* = 29)	0	0	17	0	0
				IQR 16–18		
	Environmental (*n* = 30)	0	0	16	0	0
				IQR 15–18		
	ATCC 27853	0	0	16	0	0
***Klebsiella pneumoniae***	Carbapenem-susceptible (*n* = 30)	0	0	19.5	7	0
				IQR 18–20	IQR 7–7	
	KPC (*n* = 30)	0	0	20	7	0
				IQR 19–22	IQR 7–7	
	ATCC 43816	0	0	19	7	0
***Enterococcus faecalis***	(*n* = 30)	0	0	16	10	13
				IQR 15–16	IQR 10–11	IQR 12–13
	NCTC 775	0	0	20	11	13
***Staphylococcus aureus***	MSSA (*n* = 20)	0	0	31	16	13
				IQR 28–31.5	IQR 15.5–16.5	IQR 13–14
	MRSA (*n* = 20)	0	0	31.5	16	14
				IQR 30–35	IQR 15–17	IQR 14–14.5
	NCTC 6571	0	0	30	15	13

DDAC = didecyldimethylammonium chloride; IACs = isopropanol + ammonium compounds; MRSA = methicillin-resistant *S. aureus*; MSSA = methicillin-susceptible *S. aureus*; KPC = carbapenem-resistant *K. pneumoniae*.
